# Non-Random Distribution of Reciprocal Translocation Breakpoints in the Pig Genome

**DOI:** 10.3390/genes10100769

**Published:** 2019-09-30

**Authors:** Brendan Donaldson, Daniel A. F. Villagomez, Tamas Revay, Samira Rezaei, W. Allan King

**Affiliations:** 1 Department of Biomedical Sciences, University of Guelph, Guelph, ON N1G 2W1, Canada; bdonalds@uoguelph.ca (B.D.); samira@uoguelph.ca (S.R.); 2 Departamento de Produccion Animal, Universidad de Guadalajara, Zapopan 44100, Mexico; dvillago@uoguelph.ca; 3 Alberta Children’s Hospital Research Institute (ACHRI), University of Calgary, Calgary, AB T2N 1N4, Canada; 4 Karyotekk Inc. Box 363 OVC, University of Guelph, Guelph, ON N1G 2W1, Canada

**Keywords:** reciprocal translocation, chromosome rearrangement, cytogenetics, pig

## Abstract

Balanced chromosome rearrangements are one of the main etiological factors contributing to hypoprolificacy in the domestic pig. Amongst domestic animals, the pig is considered to have the highest prevalence of chromosome rearrangements. To date over 200 unique chromosome rearrangements have been identified. The factors predisposing pigs to chromosome rearrangements, however, remain poorly understood. Nevertheless, here we provide empirical evidence which sustains the notion that there is a non-random distribution of chromosomal rearrangement breakpoints in the pig genome. We sought to establish if there are structural chromosome factors near which rearrangement breakpoints preferentially occur. The distribution of rearrangement breakpoints was analyzed across three level, chromosomes, chromosome arms, and cytogenetic GTG-bands (G-banding using trypsin and giemsa). The frequency of illegitimate exchanges (e.g., reciprocal translocations) between individual chromosomes and chromosome arms appeared to be independent of chromosome length and centromere position. Meanwhile chromosome breakpoints were overrepresented on some specific G-bands, defining chromosome hotspots for ectopic exchanges. Cytogenetic band level factors, such as the length of bands, chromatin density, and presence of fragile sites, were associated with the presence of translocation breakpoints. The characteristics of these bands were largely similar to that of hotspots in the human genome. Therefore, those hotspots are proposed as a starting point for future molecular analyses into the genomic landscape of porcine chromosome rearrangements.

## 1. Introduction

Chromosome rearrangements are known to be one of the main etiological factors contributing to hypoprolificacy in domestic species, especially the domestic pig [1,2,3]. To date over 200 distinct chromosome rearrangements have been identified in the domestic pig. The vast majority are balanced reciprocal translocations, making up over 90% of described rearrangements [4]. It is estimated that chromosome rearrangements in domestic pigs occur spontaneously in one of 200 live births [5,6]. The prevalence of chromosome rearrangements among swine herds is thought to be between 0.5% and 1.5%, dependent on the intensity of cytogenetic screening within these populations [3,5,6]. Although reciprocal translocations are quite prevalent throughout swine herds and the genetic and genomic reasons for this are poorly understood.

Reciprocal translocations are rearrangements involving two non-homologous chromosomes which break simultaneously, and subsequently misrepair, resulting in an exchange of chromosome segments. Research that involves the human genome has suggested that translocation breakpoints occurred nonrandomly for each individual chromosome pair, with specific chromosomes and cytogenetic landmarks being particularly susceptible to breakage. Various chromosomal features recognizable in a karyotype such as the total length of chromosomes and chromosome arms, chromosome morphology, as well as, chromatin density (heterochromatic and euchromatic), and the presence of common fragile sites have all been suggested to influence the frequency at which chromosome regions rearrange [7,8,9,10].

Despite the considerable number of chromosome rearrangements identified in the domestic pig, the characteristics of these rearrangements is largely unknown. Some work suggests that there is a non-random distribution of translocation breakpoints across chromosomes in the pig genome [1,11]. Amongst the first five reciprocal translocations identified in pigs, 2 breakpoints appeared on chromosome 6, and three breakpoints were found on chromosome 14 [11,12,13,14]. The presence of multiple breakpoints in close proximity on chromosome 14 led to the suggestion that regions of fragility promoting chromosome breakage may be present in the pig karyotype, and that there may be a non-random distribution of translocation breakpoints [14,15]. In addition, many breakpoints are known to overlap with common fragile sites, regions of the chromosomes which are susceptible to breakage under exposure of specific chemical stressors [16].

Although a handful of chromosomes have been examined in some detail, no comprehensive analysis of translocation breakpoints across the pig karyotype has ever been conducted. The identification of chromosome rearrangements in swine herds has increased in the past two decades with the continuation of a large screening programs at the National Veterinary School of Toulouse in France, and at the University of Guelph in Canada. With over 190 unique reciprocal chromosome translocations identified in the domestic pig, it is now easier to observe patterns in the number of breakpoints on chromosomes, chromosome arms, and cytogenetic landmarks. 

Using 195 reciprocal chromosome rearrangements identified in our lab and those reported in the literature we performed a comprehensive analysis of the translocation breakpoints at the whole chromosome and cytogenetic band levels. Our observation of rearrangements breakpoints lead us to add empirical evidence that breakpoints are nonrandomly distributed across chromosomes, and cytogenetic bands.

## 2. Materials and Methods

### 2.1. Cytogenetic Screening Analysis of Pig Populations

Peripheral blood samples were routinely collected from 5802 reproductively unproven young boars raised at various Canadian farms by experienced farm workers or Canadian Food Inspection Agency veterinarians according to the Canadian Council on Animal Care and the University of Guelph’s Animal Care Committee guidelines. These animals were from commercial herds, in good general health. The samples were submitted to the Animal Health Laboratory of the University of Guelph for commercial genetic screening. Data from this analysis was provided for use in this study. Lymphocyte cultures were set up according to the standard cytogenetic protocols of our laboratory, as previously published [6,17]. Twenty-five metaphases were captured from each animal and a minimum of two optimal quality GTG (G-banding using trypsin and giemsa)-banded karyotypes were arranged at the level of 400 bands resolution [18]. Following this conventional procedure, we identified 29 constitutional reciprocal chromosome translocations (Table S1).

### 2.2. Selection and Analysis of Reciprocal Chromosome Translocations Published in the Literature

A comprehensive list of G-banded reciprocal chromosome translocations, counting the 29 reciprocal chromosome translocations identified in our own lab and by updating a previously published list of G-banded reciprocal chromosome translocations [4], resulted in a total of 195 unique rearrangements (Table S1). Breakpoints on sex chromosomes were not included in the analysis due to the rarity of such rearrangements relative to autosomal chromosomes.

### 2.3. Definition of Chromosome Parameters

The physical length in megabases (Mb) for each chromosome was obtained from the 11.1 Sus Scrofa genome assembly (https://www.ncbi.nlm.nih.gov/genome/84). Using the physical lengths of chromosomes as a basis, the lengths of cytogenetic bands were estimated as follows. The standard GTG-banded ideogram and chromosome landmarks of the domestic pig karyotype were used as a reference to measure and calculate the fractional lengths of each cytogenetic band per chromosome [18] (Figure S1). The physical length of each band, and their start and stop points were calculated by multiplying the fractional length with the physical length of the chromosome (Table S2). This resulted in a conversion map between cytogenetic bands and their physical length. 

These measurements were verified by selecting 25 bacterial artificial chromosome (BAC) clones from the literature that were FISH mapped to cytogenetic bands, as well as physically mapped to exact genetic loci in the genome (Table S3). These cytogenetic bands were then converted by our map to physical positions and compared to the established DNA positions. Of the 25 cytogenetically mapped probes, 20 fell within the estimated physical positions for their respective cytogenetic bands, and the remaining five fell within an adjacent band. Therefore, it was assumed that the method for estimating pig cytogenetic band lengths is sufficient for rough estimations (within approximately 3 million base pairs).

We defined the translocation frequency of each chromosome segment (i.e., whole chromosome, chromosome arm, cytogenetic band, and groups of chromosomes) as the number of translocation breakpoints per 1 Mb. The translocation frequency was, thus, calculated as the number of translocation breakpoints for a given chromosome segment, over the physical length of the chromosome segment, resulting in the number of translocation breakpoints per 1 Mb of chromosome material. The expected number of translocations per chromosome segment was calculated by multiplying the total number of breakpoints by the chromosome segment length (Table S2). 

The standard GTG-banded karyotype of the domestic pig was used to define each cytogenetic band, and their GTG-banding designation [18] (Figure S1). In total there were 267 distinct cytogenetic bands across the 18 autosomal chromosomes. The positions of cytogenetic bands on chromosome arms were defined as proximal, median, and distal according to the position of the band in the top third, middle third, and bottom third of bands, respectively on each chromosome [18,19]. A list of common fragile sites in the pig genome was used to define which bands had a common fragile site [16] (Table S4).

### 2.4. Statistical Analysis

Statistical analysis was performed in R 3.5.1 [20]. Spearman’s rank correlation coefficient was applied in order to determine the presence of an association between two variables. The Chi-square test was applied in order to determine a statistical difference between the observed and expected frequencies of variables such as translocation breakpoint number. The Student’s *t*-test was used in order to determine if the means of two groups were significantly different from one another. One-way analysis of variance (ANOVA) was used to determine if the means of three or more groups were significantly different from one another. The Poisson distribution was used to determine if translocation breakpoints occurred on cytogenetic bands independently of one another. 

## 3. Results

### 3.1. The Translocation Frequency of Chromosomes is Independent of Physical Length

**Figure 1:**
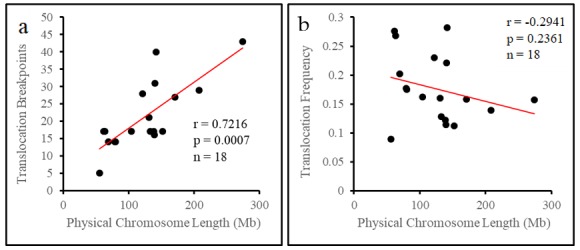
Physical chromosome length is associated with the number of breakpoints on chromosomes, but not the translocation frequency. (**a**) Scatterplot comparing the number of breakpoints to the physical length of each chromosome. Spearman’s correlation coefficient was used to determine if there is a relationship between the two variables, *r* = Spearman’s correlation coefficient, *p* = numerical representation that the result was seen by chance, *n* = number of chromosomes considered in the analysis. (**b**) Scatterplot comparing the translocation frequency and physical length of each chromosome. Spearman’s correlation coefficient was used as described above.

A total of 195 unique reciprocal chromosome translocations were considered for this study (Table S1). We mapped all translocation breakpoints to the pig karyotype and summed the number of breakpoints on each chromosome (Table 1). The number of breakpoints per chromosome ranged between 43 on chromosome 1, and 5 on chromosome 18, with longer chromosomes appearing to have more breakpoints than shorter chromosomes. We considered if there was a correlation between physical chromosome length and breakpoints. Unsurprisingly, we found that translocation breakpoints preferentially occur on longer chromosomes (*r* = 0.722, *p* = 0.0007, Spearman’s correlation coefficient, Figure 1a). Chromosomes, however, generally did not break in proportion to their length, with many chromosomes having a significant difference between their observed and expected breakpoint number (X^2^ = 35.99, *p* = 0.0046, Chi-square test, Table 1). Many chromosomes had a greater than 10% difference between observed and expected breakpoints, which on average worked out to a difference of five breakpoints. This difference appeared to be independent of the length of the chromosomes.

To better compare chromosomes of different lengths we calculated the translocation frequency (breakpoints per Mb) for each chromosome (Table 1). The translocation frequencies of chromosomes appeared to complement the difference between observed and expected breakpoints as those chromosomes with more breakpoints than expected had higher translocation frequencies than chromosomes with fewer breakpoints than expected. The translocation frequency of chromosomes appeared to be unrelated to physical length. Comparing the translocation frequency and physical chromosome length, we found no correlation between the two (*r* = −0.294, *p* = 0.236, Spearman’s correlation test, Figure 1b). Thus, we see through observing the translocation frequency that shorter chromosomes may have high densities of breakpoints, and that this appears to not influence chromosome length. As such, once length is controlled for and longer chromosomes do not appear more prone to breakage than shorter chromosomes.

### 3.2. The Translocation Frequency of Chromosome Arms is Independent of Physical Length

**Figure 2:**
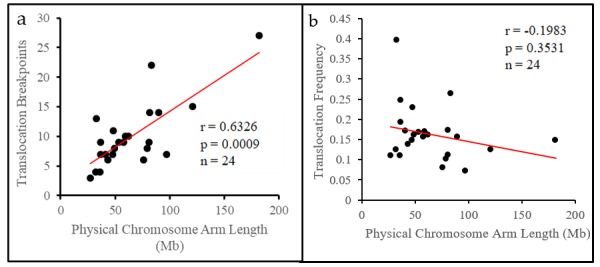
Physical length of chromosome arms is associated with breakpoint number but not the translocation frequency of chromosome arms. (**a**) Scatterplot comparing the number of breakpoints to the physical length of each chromosome arm. Spearman’s correlation coefficient was used to determine if there is a relationship between the two variables. r = Spearman’s correlation coefficient, *p* = numerical representation that the result was seen by chance, and n = number of chromosome arms included in the analysis. (**b**) Scatterplot comparing the translocation frequency and physical length of each chromosome arm. Spearman’s correlation coefficient was used as described above.

Sus Scrofa chromosomes, chromosome 1 through chromosome 12 present with distinct chromosome arms (e.g., biarmed chromosomes), and have a variable number of translocation breakpoints between their short (p arm) and long arms (q arm). We compared the physical length of each biarmed chromosome to their breakpoint number (Table 2), observing that translocation breakpoints preferentially occurred on longer chromosome arms (*r* = 0.632, *p* = 0.0009, Spearman’s correlation test, Figure 2a). Chromosome arms were generally found to rearrange in proportion to their length, with an average difference of only 2.5 breakpoints between the observed and expected number (X^2^ = 34.797, df = 23, *p* = 0.055, Chi-square test, Table 2).

Controlling for length via the translocation frequency revealed that most chromosome arm pairs (p and q) had different translocation frequencies even though they were part of the same chromosome. The average difference in translocation frequency between p and q arms for each chromosome was 64.3%, or 0.08 translocations per Mb (Table 3). The difference in translocation frequency between p and q arms was independent of length (*r* = −0.198, *p* = 0.353, Spearman’s correlation test, Figure 2b). The translocation frequency of chromosome arms is, therefore, independent of physical length, and chromosome arm pairs rearrange independently of one another. 

### 3.3. Translocation Breakpoints Preferentially Occur on Longer Cytogenetic Bands

**Figure 3:**
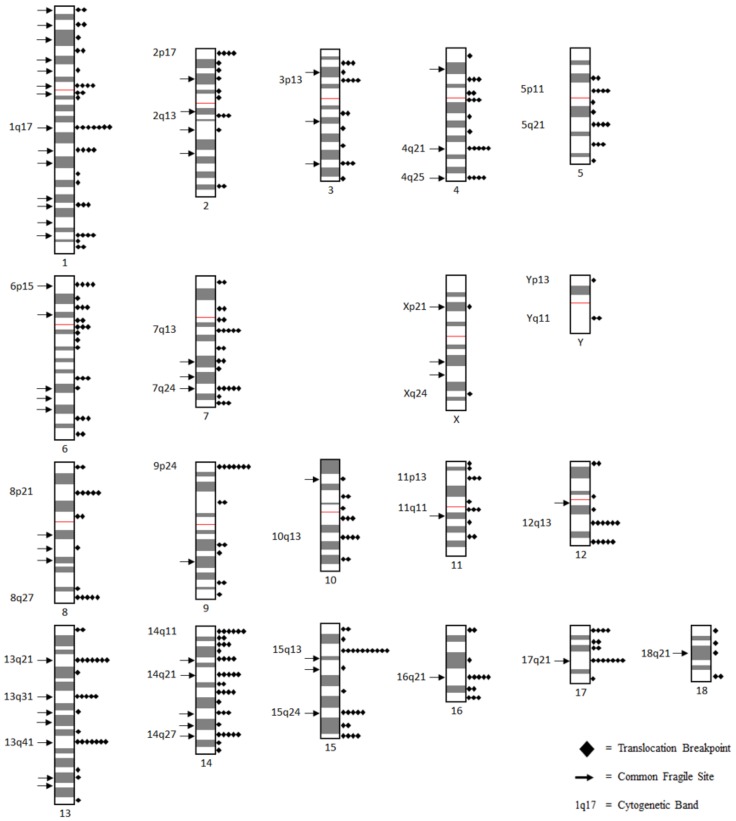
Ideogram of the domestic pig karyotype with important cytogenetic markers displayed. An ideogram of the standard GTG(G-banding using trypsin and giemsa)-banded karyotype of the domestic pig is displayed. Diamonds represent breakpoints on cytogenetic bands. Arrows indicate bands with known common fragile sites. Cytogenetic bands on each chromosome are pointed out, usually those bands with the most breakpoints, to help the positional context of each chromosome.

**Figure 4:**
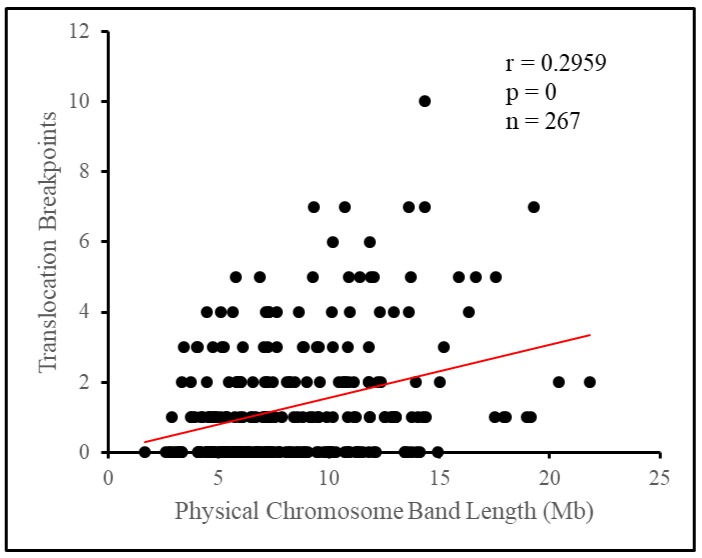
Physical cytogenetic band length is associated with the number of translocation breakpoints. (a) Scatterplot comparing the number of breakpoints to the physical length of each cytogenetic band. Spearman’s correlation coefficient was used to determine if there is a relationship between the two variables. *r* = Spearman’s correlation coefficient and *p* = numerical representation that the result was seen by chance.

In total 352 defined autosomal cytogenetic breakpoints were considered for band level analysis. We mapped these breakpoints onto the standard GTG-banded pig karyotype, denoting the number of breakpoints per band (Figure 3; Figure S1). The distribution of breakpoints appeared uneven, with the number of breakpoints per band ranging between zero and ten. The number of breakpoints per band did not fit a Poisson distribution. Many more bands than expected had no observed breakpoints or had four or more breakpoints, while there was a deficiency of bands with one to three breakpoints (X^2^ = 388.33, *p* < 1 × 10^−5^, Chi-square test, Table 4). Given that length is a known factor that influences breakpoint number on chromosomes, we considered if the length of bands may be related to this discrepancy. Comparing the estimated physical length of bands (Table S2) to their breakpoint number we found a slight yet significant correlation between the two (*r* = 0.296, *p* = 1 × 10^−5^, Spearman’s correlation test, Figure 4), indicating that longer bands tended to have more breakpoints.

### 3.4. Translocation Breakpoints Do Not Preferentially Occur on Specific Chromosomal Positions

We considered whether the relative position of a cytogenetic band on chromosome arms affected its translocation frequency. Cytogenetic bands were defined as proximal, median, and distal (see Methods). Each group of bands was found to rearrange in proportion to their total length (X^2^ = 0.843, *p* = 0.656, Chi-square test, Table 5). We then compared the translocation frequencies of the bands of each group, finding no relationship between the position of a band on a chromosome arm and translocation frequency of bands (*p* = 0.707, one-way Anova, Table 5). Therefore, a band’s position on the chromosome arm relative to the centromere had no apparent influence over translocation frequency. 

### 3.5. Translocation Breakpoints Preferentially Occur on G-Negative Bands

Observing the distribution of breakpoints in the G-banded pig karyotype it appeared that G-negative bands had the larger concentration of breakpoints. Although G-negative and G-positive bands make up a similar proportion of cytogenetic bands (55% to 44%), translocation breakpoints appeared to preferentially occur on G-negative bands, with 87% of breakpoints occurring in such bands (Table 6). The translocation frequency of G-negative and G-positive bands were compared and showed that G-negative bands generated translocation breakpoints at five times the frequency of G-positive bands (*t* = 8.87, *p* < 1 × 10^−5^, Student’s *t*-test). Thus, we observe that breakpoints appeared to preferentially occur on G-negative bands. 

### 3.6. Translocation Breakpoints Preferentially Occur on Cytogenetic Bands with Fragile Sites

Common fragile sites are known to overlap with cytogenetic breakpoints in the pig genome. We considered 57 autosomal common fragile sites, across the pig genome, and referred to any cytogenetic band with a fragile site as a fragile band (see Methods; Table S4). Fragile bands make up only 20.6% of bands, however, have 33.2% of breakpoints (Table 7). Normalizing for length by calculating expected values for translocation breakpoints, we see that fragile bands have far more translocation breakpoints than would otherwise be expected (X^2^ = 6.459, *p* =0.011, Chi-square test, Table 7). The translocation frequency of fragile bands exemplified this difference as fragile bands had a translocation frequency 35% higher than normal bands (*t* = −1.792, *p* = 0.037, Student’s *t*-test). Notably, not all fragile bands appeared to translocate more frequently. Many fragile bands had no or very few breakpoints, however 17 of the 57 bands had very high translocation frequencies, over twice the average of cytogenetic bands in general, and appeared to drive the relationship between fragile bands and higher translocation frequency. 

### 3.7. Translocation Breakpoints Preferentially Occur on G-negative Bands with Fragile Sites

Given that some fragile bands appeared to translocate more often than others we divided bands into groups based on the presence of a fragile site and the G-banding pattern. We compared the observed and expected breakpoint numbers of each group, finding that both G-negative groups had more breakpoints than expected, while both G-positive groups had fewer (X^2^ = 138.481, *p* < 1 × 10^−5^, Chi-square test, Table 8). The translocation frequency of the cytogenetic bands within each group were compared and showed a significant difference between the groups (*p* < 1 × 10^−5^, one-way Anova, Table 8). G-negative-fragile bands had the highest translocation frequency, followed by G-negative-normal bands, while both G-positive groups had similarly low translocation frequencies. The translocation frequencies of G-negative-fragile and G-negative-normal bands were compared, and found to be significantly different, indicating that G-negative bands with fragile sites translocated the most frequently of all cytogenetic bands (*t* = 2.046, *p* = 0.021, Student’s *t*-test). This suggests that the presence of a common fragile site may only influence translocation frequency of G-negative bands. 

We performed multiple linear regression analysis to establish how the length of cytogenetic bands, G-banding, and fragility influence breakpoint number and translocation frequency. We found that all three variables significantly contributed to a linear model of breakpoint number on cytogenetic bands, with G-banding being the best predictor of breakpoint number (*p* < 2 × 10^−16^, Table 9). The adjusted R^2^ value, however, showed that these three variables only explained 31.9% of the variation in breakpoint number amongst cytogenetic bands. Considering translocation frequency of bands, G-banding was found to be highly associated with translocation frequency of cytogenetic bands, while fragility was not. These two variables, however, explained only 22.7% of the variation in translocation frequency present on cytogenetic bands (adjusted R^2^ = 0.221, Table 9). Overall, we observe that G-banding, physical length, and the presence of fragile sites only moderately explain the number of breakpoints on cytogenetic bands.

## 4. Discussion and Conclusions

Observing the distribution of translocation breakpoints across chromosomes, chromosome arms, and cytogenetic bands revealed the chromosome regions most susceptible to rearrangement, and physical features that are associated with higher breakpoint number and translocation frequency. Translocation breakpoints are nonrandomly distributed across chromosomes and chromosome arms, with particular chromosomes appearing to be far more susceptible to rearrangement than others. In addition, the length of cytogenetic bands, G-banding (heterochromatin and euchromatin), and the presence of fragile sites were all found to be associated with a higher number of breakpoints on cytogenetic bands. In particular we observed that G-negative bands had high translocation frequencies on average, with those G-negative bands with common fragile sites having the highest translocation frequencies of all bands on average. 

Taking the chromosome as an individual unit, we found that chromosomes do not rearrange in direct proportion to their length. Although longer chromosomes tended to have more breakpoints in general, length appeared to have no relationship with whether chromosomes had a deficiency or a surplus of breakpoints. The physical length of chromosomes has long been suggested to influence the number of translocation breakpoints on each chromosome in the pig [4,21], and human [9,22] genomes. The rationale behind this is that longer chromosomes should have more opportunities for breakage, and therefore should break and translocate more often. Taken simply as a numerical value, this is generally true in the pig, however once length is accounted for and we see no evidence that longer physical length is associated with higher translocation frequency. Both long and short chromosomes have high (chromosomes 12 and 14) and low (chromosomes 2 and 18) translocation frequencies. Those chromosomes with the highest translocation frequencies appear to have some feature that promotes more frequent rearrangement. This suggests that chromosome features beyond the simple physical length and breakpoint number should be considered for potential roles in promoting translocation events. 

Observing the number of breakpoints relative to the physical length of chromosome arms yielded slightly different results. Longer chromosome arms tended to have more translocation breakpoints, and chromosome arms typically rearranged in proportion to their length. As with whole chromosomes, longer chromosome arms have been previously suggested to be predisposed to having more translocation breakpoints [10]. Examining the translocation frequencies of the p and q arms of each chromosome, however, revealed a different trend. We observed that these chromosome arm pairs may have considerably different translocation frequencies from one another even though they are part of the same chromosome, for example, chromosome arm 12q has a translocation frequency 3.4x higher than chromosome arm 12p. Differences in breakpoint number between chromosome arm pairs has been previously established [8], however this difference in translocation frequency is perhaps more unexpected, as length is taken into account, and suggests that the factors that promote rearrangement on each chromosome are not necessarily present across the entire chromosome, and may be more localized to specific regions.

Breaking the chromosome down further into cytogenetic bands, we observed the appearance of a non-random distribution of breakpoints. Attempting to fit the number of breakpoints per band under a Poisson distribution yielded a poor fit, as a surplus of bands had no breakpoints at all, or four or more breakpoints, while there was a deficiency of bands with one to three breakpoints. As such, there tended to be a clustering of breakpoints on relatively few bands, with 12.4% of bands, those with four or more breakpoints, having 47.7% of all breakpoints between them. Length appeared to influence some of this difference as longer bands tended to have more breakpoints, however, this influence appeared small overall. Translocation breakpoints in the pig genome have previously been suggested to be distributed nonrandomly based on empirical evidence [15]. In the human genome, breakpoints have previously been shown to be nonrandomly distributed across cytogenetic bands, with clusters of breakpoints on individual bands being referred to as hotspots for rearrangement [7,9,10,23]. Clustering of breakpoints in the pig and human genomes occur in a variety of positions, with no apparent influence due to the relative position to the centromere on the chromosome arm [24]. The clustering of breakpoints in a few cytogenetic bands in distinct chromosomal positions, such as 1q17 and 15q13, suggests that individual bands may have some feature that promotes rearrangement events. 

We found that G-negative bands were strongly associated with higher breakpoint numbers and translocation frequency. G-negative bands as a whole had a translocation frequency five times greater than that of G-positive bands. These results are in agreement with several studies of translocation breakpoints in humans which consistently indicate that cytogenetic G-negative bands have a higher density of breakpoints than G-positive bands [7,8,9,10]. The more open chromatin composition of these bands is proposed to be more susceptible to breakage than more condensed regions of chromosomes, although it has been suggested that cytogeneticists may be biased towards placing rearrangements within G-negative bands due to the contrasting nature of light and dark bands. Nevertheless, studies of R-banded chromosomes indicate that most rearrangements are identified in R-positive (G-negative) bands [24,25]. Therefore, our findings provide more evidences that sustain the notion that euchromatic chromosome regions are more susceptible to breakage and subsequent rearrangement. 

The presence of common fragile sites within G-negative bands was found to be associated with higher translocation frequency. In contrast the presence of common fragile sites in G-positive bands appeared to have no influence on translocation frequency. Common fragile sites in the pig karyotype have been previously noted to overlap with the cytogenetic positions of translocation breakpoints [6,16,26,27]. Our results are generally consistent with those found in humans, as cytogenetic bands with fragile sites in the human genome were found to translocate more frequently than bands without fragile sites [9,28,29,30]. These results suggest that the presence of common fragile sites on G-negative bands may influence rearrangement by promoting breakage on more open chromatin regions which already appear more susceptible to rearrangement by their nature. 

Considering the features associated with breakpoint number, we determined that the chromatin density (e.g., type of banding) followed by length of bands, and the presence of common fragile sites were found to significantly contribute to a regression model of translocation breakpoint number. Only chromatin density contributed to a model of translocation frequency, however, which is in line with the presence of common fragile sites only influencing translocation frequency when present in G-negative bands. Chromatin density and physical chromosome length have previously been shown to influence breakpoint number in the human genome [10,22], however, little work has been done to demonstrate if these factors influence translocation frequency in the human genome. Although it is apparent that length, chromatin density, and the presence of fragile sites play a role in influencing breakpoint number, together at most they explain approximately a third of the variation present amongst bands. We may speculate then that more specific chromosome features, unique to each band, may contribute more specifically to the promotion of translocation events. 

We determined that translocation breakpoints appeared to be nonrandomly distributed across cytogenetic bands, and that factors such as length, G-banding, and the presence of fragile sites were all related to breakpoint number. We thus sought to propose hotspots for chromosome rearrangement in the pig genome based on these characteristics. Starting with the average number of breakpoints per band, 2.4, and average translocation frequency, 0.29 per Mb, we proposed that any band that had at least five breakpoints and/or a translocation frequency of 0.58 per Mb or higher to be hotspots for rearrangement in the pig genome. In total, nineteen bands based on number of breakpoints (Table 10), and fifteen bands based on translocation frequency (Table 11) were proposed as hotspots for rearrangement. Six bands were shared between the lists. These bands are derived from a variety of chromosomes and positions. All bands are in G-negative regions, and twelve had a common fragile site. Notably, many bands from shorter chromosomes with high translocation frequencies feature prominently amongst our proposed hotspot bands. For instance, chromosome 12, with just seventeen breakpoints has three bands amongst our hotspots, indicating the specific bands on this chromosome that appear to drive the high translocation frequency of this chromosome. In total, thirty bands with varied characteristics were proposed as hotspots for rearrangement in the pig genome.

For the first time, all known porcine reciprocal translocations were analyzed, revealing the chromosomes and cytogenetic bands with the highest number of breakpoints and highest translocation frequencies. The cytogenetic bands with the highest number of breakpoints and highest translocation frequencies were selected as potential hotspots for rearrangement in the pig karyotype. This work can serve as a foundation for future endeavors to assist breeders in the selection of pigs that are less susceptible to chromosome rearrangement, ultimately increasing efficiency and profitability for breeders. The comparison of chromosome rearrangements in the pig and human genomes suggests that the pig may serve as an appropriate biomedical model to study chromosome rearrangements. Although less than 200 porcine translocations were considered, this is the most complete analysis of chromosome rearrangements in the pig and it provides a baseline for future considerations of porcine chromosome rearrangements. Given the analyses concerning chromosome features easily mappable to the pig karyotype, future considerations should be given to the specific molecular and genomic landscape present on cytogenetic bands, in order to characterize which factors promote rearrangement, and explain why specific cytogenetic bands may have more breakpoints or higher translocation frequencies than other bands.

## Figures and Tables

**Table 1:** Distribution of translocation breakpoints across chromosomes.

Chromosome	Observed Breakpoints	Physical Chromosome Length (Mb)	Expected Breakpoints	X^2^ Value	Fold Change Between Observed and Expected	Translocation Frequency
1	43	274.331	46.49	0.262	0.9249	0.1567
2	17	151.936	25.75	2.9733	0.6602	0.1119
3	17	132.849	22.52	1.353	0.7549	0.128
4	21	130.911	22.19	0.0638	0.9464	0.1604
5	17	104.526	17.71	0.0285	0.9599	0.1626
6	27	170.844	28.95	0.1313	0.9326	0.158
7	28	121.844	20.65	2.6161	1.3559	0.2298
8	17	138.966	23.55	1.8218	0.7219	0.1223
9	16	139.512	23.64	2.4691	0.6768	0.1147
10	14	69.359	11.75	0.4309	1.1915	0.2018
11	14	79.17	13.42	0.0251	1.0432	0.1768
12	17	61.603	10.44	4.122	1.6284	0.276
13	29	208.335	35.31	1.1276	0.8213	0.1392
14	40	141.755	24.02	10.6312	1.6653	0.2822
15	31	140.413	23.8	2.1782	1.3025	0.2208
16	14	79.944	13.55	0.0149	1.0332	0.1751
17	17	63.494	10.76	3.6187	1.5799	0.2677
18	5	55.983	9.49	2.1244	0.5269	0.0893
X^2^ = 35.992, d.f = 17, *p* = 0.0046, σ_est_ = 6.389						

**Table 2:** The distribution of translocation breakpoints across chromosome arms.

Chromosome Arm	Observed Breakpoints	Physical Chromosome Arm Length (Mb)	Expected Breakpoints	X^2^ Value	Fold Change Between Observed and Expected	Translocation Frequency
1p	14	90.049	13.84	0.0018	1.0116	0.1555
2p	9	53.556	8.23	0.072	1.0936	0.168
3p	8	49.508	7.61	0.02	1.0512	0.1616
4p	7	47.557	7.31	0.0131	0.9576	0.1472
5p	6	43.588	6.7	0.0731	0.8955	0.1377
6p	11	48.264	7.42	1.7273	1.4825	0.2279
7p	4	36.398	5.59	0.4523	0.7156	0.1099
8p	9	57.667	8.86	0.0022	1.0158	0.1561
9p	10	62.169	9.55	0.0212	1.0471	0.1609
10p	4	32.049	4.92	0.172	0.813	0.1248
11p	7	36.45	5.6	0.35	1.25	0.192
12p	3	27.225	4.18	0.3331	0.7177	0.1102
1q	27	182.052	27.97	0.0336	0.9653	0.1483
2q	7	97.05	14.91	4.1964	0.4695	0.0721
3q	9	81.3	12.49	0.9752	0.7206	0.1107
4q	14	81.347	12.5	0.18	1.12	0.1721
5q	10	59.272	9.11	0.0869	1.0977	0.1687
6q	15	120.939	18.58	0.6898	0.8073	0.124
7q	22	83.492	12.83	6.5541	1.7147	0.2635
8q	8	79.19	12.17	1.4288	0.6574	0.101
9q	6	75.953	11.67	2.7548	0.5141	0.079
10q	9	36.428	5.6	2.0643	1.6071	0.2471
11q	7	40.953	6.29	0.0801	1.1129	0.1709
12q	13	32.889	5.05	12.5153	2.5743	0.3953
X^2^ = 34.797, d.f = 23, *p* = 0.0545,σ_est_ = 3.66						

**Table 3:** Translocation frequency of chromosome arms.

Chromosome	P Arm Translocation Frequency	Q Arm Translocation Frequency	Percent Change in Translocation Frequency Between Q and P arms
1	0.1555	0.1483	−4.63%
2	0.168	0.0721	−57.08%
3	0.1616	0.1107	−31.5%
4	0.1472	0.1721	16.92%
5	0.1377	0.1687	22.51%
6	0.2279	0.124	−45.59%
7	0.1099	0.2635	139.76%
8	0.1561	0.101	−35.3%
9	0.1609	0.079	−50.9%
10	0.1248	0.2471	97.99%
11	0.192	0.1709	−10.99%
12	0.1102	0.3953	258.71%

**Table 4:** Distribution of cytogenetic bands that possess a given number of translocation breakpoints.

Number of Breakpoints per Band	Number of Cytogenetic Bands		Fold Change Between Observed and Expected
	Observed	Expected ^a^	
0	121	71.32	1.6966
1	60	94.15	0.6373
2	35	62.13	0.5633
3	18	27.34	0.6584
4	14	9.02	1.5521
5	11	2.38	4.6218
6	2	0.52	3.8462
7+	6	0.12	50
^a^ Based on a Poisson Distribution with m = 1.32 and n = 267, X^2^ = 388.333, d.f = 7, *p* < 1 × 10^−5^, σ_est_ = 23.692			

**Table 5:** The distribution of breakpoints by chromosomal position.

G-Banding	Number of Cytogenetic Bands	Physical Length of Cytogenetic Bands (Mb)	Observed Breakpoints	Expected Breakpoints	X^2^ Value	Fold Change Between Observed and Expected	Translocation Frequency
Proximal	91	739.493	119	115.93	0.0813	1.0265	0.1609
Median	78	778.896	114	122.11	0.5386	0.9336	0.1464
Distal	98	726.879	119	113.96	0.2229	1.0442	0.1637
X^2^ = 0.843, d.f = 2, *p* = 0.656, σ_est =_ 5.791							

**Table 6:** Comparison of translocation breakpoints on G-negative and G-positive cytogenetic bands.

G-Banding	Number of Cytogenetic Bands	Physical Length of Cytogenetic Bands (Mb)	Observed Breakpoints	Expected Breakpoints	X^2^ Value	Fold Change Between Observed and Expected	Translocation Frequency
G-negative	148	1271.776	305	199.38	55.9514	1.5297	0.2398
G-positive	119	973.492	47	152.62	73.0939	0.308	0.0483
X^2^ = 129.045, d.f = 1, *p* < 1 × 10^−5^, σ_est =_ 105.62							

**Table 7:** Comparison of translocation breakpoints on cytogenetic bands with common fragile sites and normal bands.

Fragility	Number of Cytogenetic Bands	Physical Length of Cytogenetic Bands (Mb)	Observed Breakpoints	Expected Breakpoints	X^2^ Value	Fold Change Between Observed and Expected	Translocation Frequency
Normal	212	1634.322	235	256.22	1.7574	0.9172	0.1438
Fragile	55	610.946	117	95.78	4.7013	1.2215	0.1915
X^2^ = 6.459, d.f = 1, *p* = 0.011, σ_est =_ 21.22							

**Table 8:** Comparison of translocation breakpoints on cytogenetic bands divided by banding and fragility.

Category	Number of Cytogenetic Bands	Length of Cytogenetic Bands (Mb)	Observed Breakpoints	Expected Breakpoints	X^2^ Value	Fold Change Between Observed and Expected Breakpoints	Translocation Frequency
G-negative-Normal	114	902.468	197	141.48	21.7873	1.3924	0.2183
G-negative-Fragile	34	369.308	108	57.9	43.3508	1.8653	0.2924
G-positive-Normal	96	731.854	38	114.74	51.325	0.3312	0.0519
G-positive-Fragile	23	241.638	9	37.88	22.0183	0.2376	0.0372
X^2^ = 138.481, d.f = 3, *p* < 0.00001, σ_est_ = 55.488							

**Table 9:** Multiple linear regression analysis for translocation breakpoints and translocation frequency.

Model: Observed Translocation Breakpoints on Cytogenetic Bands				
Variable	Coefficient	Standard Error	*t* Value	*p* Value
Banding	1.5976	0.1781	8.972	< 2 × 10^−16^
Physical Length (Mb)	0.1221	0.0247	4.941	1.38 × 10^−06^
Fragility	0.4893	0.2275	2.15	0.0324
Model Summary: N = 267, R^2^ = 0.3267, Adjusted R^2^ = 0.3191, *p* < 2.2 × 10^−16^				
**Model: Translocation Frequency of Cytogenetic Bands**				
**Variable**	**Coefficient**	**Standard Error**	***t*** **Value**	***p*** **Value**
Banding	0.1928	0.0224	8.599	4.335 × 10^−16^
Fragility	0.0408	0.0272	1.501	0.1346
Model Summary: N = 267, R^2^ = 0.2269, Adjusted R^2^ = 0.2211, *p* = 1.759 × 10^−15^				

**Table 10:** Suggested hotspots for rearrangement in the pig genome based on breakpoint number.

Hotspot	Observed Breakpoints	Physical Cytogenetic Band Length (Mb)	Expected Breakpoints	X^2^ Value	Fold Change Between Observed and Expected	Translocation Frequency
15q13*	10	14.326	2.25	26.6944	4.4444	0.698
1q17*^1^	7	10.738	1.68	16.8467	4.1667	0.6519
9p24*	7	14.346	2.25	10.0278	3.1111	0.4879
13q21^1^	7	13.603	2.13	11.1347	3.2864	0.5146
13q41^1^	7	19.289	3.02	5.2452	2.3179	0.3629
17q21^1^	7	9.302	1.46	21.0216	4.7945	0.7525
12q13	6	11.864	1.86	9.2148	3.2258	0.5057
14q11*	6	10.177	1.6	12.1	3.75	0.5896
4q21^1^	5	12.04	1.89	5.1175	2.6455	0.4153
7q13*	5	6.851	1.07	14.4345	4.6729	0.7298
7q24^1^	5	9.272	1.45	8.6914	3.4483	0.5393
8p21	5	16.63	2.61	2.1885	1.9157	0.3007
8q27	5	11.401	1.79	5.7565	2.7933	0.4386
12q15*	5	5.798	0.91	18.3825	5.4945	0.8624
13q31^1^	5	13.696	2.15	3.7779	2.3256	0.3651
14q21^1^	5	15.891	2.49	2.5302	2.008	0.3146
14q27^1^	5	10.904	1.71	6.3299	2.924	0.4585
15q24^1^	5	11.879	1.86	5.3009	2.6882	0.4209
16q21^1^	5	17.556	2.75	1.8409	1.8182	0.2848
X^2^ = 186.636, d.f = 18, *p* < 1 × 10^−5^, σ_est_ = 4.157, * = Band has both ≥5 breakpoints, and >0.58 translocationp frequency, ^1^ = Band has a common fragile site						

**Table 11:** Potential hotspots for rearrangement in the pig genome based on translocation frequency.

Hotspot	Observed Breakpoints	Physical Cytogenetic Band Length (Mb)	Expected Breakpoints	X^2^ Value	Fold Change Between Observed and Expected	Translocation Frequency
10q13	4	4.449	0.7	15.5571	5.7143	0.8991
11q11	3	3.413	0.54	11.2067	5.5556	0.879
12q15*	5	5.798	0.91	18.3825	5.4945	0.8624
14q15^1^	4	5.114	0.8	12.8	5	0.7822
9p24*	7	9.302	1.46	21.0216	4.7945	0.7525
4q11	3	4.013	0.63	8.9157	4.7619	0.7476
6q11	3	4.078	0.64	8.7025	4.6875	0.7357
7q13*	5	6.851	1.07	14.4345	4.6729	0.7298
15q26	4	5.628	0.88	11.0618	4.5455	0.7107
15q13*	10	14.326	2.25	26.6944	4.4444	0.698
1q17*^1^	7	10.738	1.68	16.8467	4.1667	0.6519
2q13	3	4.73	0.74	6.9022	4.0541	0.6342
12p15	2	3.347	0.52	4.2123	3.8462	0.5976
14q11*	6	10.177	1.6	12.1	3.75	0.5896
14q13	3	5.14	0.81	5.9211	3.7037	0.5837
X^2^ = 194.759, d.f =14, *p* < 1 × 10^−5^, σ_est_ = 3.784, * = Band has both ≥5 breakpoints, and >0.58 translocation frequency, ^1^ = Band has a common fragile site						

## Supplementary Materials

The following are available online at https://www.mdpi.com/2073-4425/10/10/769/s1, Table S1:List of Chromosome Rearrangements in the Pig Genome, Figure S1: Standard Karyotype of the Domestic Pig, Table S2: Estimated Physical Length of Porcine Cytogenetic Bands, Table S3: Verification of Estimated Cytogenetic Band Lengths using BAC Clones, Table S4: List of Common Fragile Sites in the Pig Genome.
